# Polymorphisms in genes implicated in dopamine, serotonin and noradrenalin metabolism suggest association with cerebrospinal fluid monoamine metabolite concentrations in psychosis

**DOI:** 10.1186/1744-9081-10-26

**Published:** 2014-07-29

**Authors:** Dimitrios Andreou, Erik Söderman, Tomas Axelsson, Göran C Sedvall, Lars Terenius, Ingrid Agartz, Erik G Jönsson

**Affiliations:** 1 Department of Clinical Neuroscience, Psychiatry Section, HUBIN Project, Karolinska Institutet and Hospital, R5:00, SE-171 76 Stockholm, Sweden; 2 Department of Medical Sciences, Molecular Medicine, Uppsala University, Uppsala, Sweden; 3 NORMENT, Institute of Clinical Medicine, University of Oslo, Oslo, Norway; 4 Department of Psychiatric Research, Diakonhjemmet Hospital, Oslo, Norway

**Keywords:** Psychosis, Schizophrenia, Psychiatric genetics, Cerebrospinal fluid, Homovanillic acid (HVA), 5-hydroxyindoleacetic acid (5-HIAA), 3-methoxy-4-hydroxyphenylglycol (MHPG)

## Abstract

**Background:**

Homovanillic acid (HVA), 5-hydroxyindoleacetic acid (5-HIAA) and 3-methoxy-4-hydroxyphenylglycol (MHPG) are the major monoamine metabolites in the central nervous system (CNS). Their cerebrospinal fluid (CSF) concentrations, reflecting the monoamine turnover rates in CNS, are partially under genetic influence and have been associated with schizophrenia. We have hypothesized that CSF monoamine metabolite concentrations represent intermediate steps between single nucleotide polymorphisms (SNPs) in genes implicated in monoaminergic pathways and psychosis.

**Methods:**

We have searched for association between 119 SNPs in genes implicated in monoaminergic pathways [tryptophan hydroxylase 1 *(TPH1), TPH2,* tyrosine hydroxylase *(TH),* DOPA decarboxylase *(DDC),* dopamine beta-hydroxylase *(DBH),* catechol-O-methyltransferase *(COMT),* monoamine oxidase A *(MAOA)* and *MAOB*] and monoamine metabolite concentrations in CSF in 74 patients with psychotic disorder.

**Results:**

There were 42 nominally significant associations between SNPs and CSF monoamine metabolite concentrations, which exceeded the expected number (20) of nominal associations given the total number of tests performed. The strongest association (p = 0.0004) was found between *MAOB* rs5905512, a SNP previously reported to be associated with schizophrenia in men, and MHPG concentrations in men with psychotic disorder. Further analyses in 111 healthy individuals revealed that 41 of the 42 nominal associations were restricted to patients with psychosis and were absent in healthy controls.

**Conclusions:**

The present study suggests that altered monoamine turnover rates in CNS reflect intermediate steps in the associations between SNPs and psychosis.

## Introduction

Schizophrenia is a disorder affecting approximately 1% of the world’s population and with heritability up to 80% [1]. Many gene variations have been associated with the disorder, however the results have been ambiguous and difficult to replicate until recently, when genome wide analyses of several thousands of individuals have identified an increasing number of loci associated with schizophrenia [2]. Measurable biological markers may form intermediate steps between gene variations and the phenotype, i.e. the psychotic disorder, and associations between the biological measures and gene variants may be more robust than the associations between gene variants and the disorder itself.

Homovanillic acid (HVA), 5-hydroxyindoleacetic acid (5-HIAA) and 3-methoxy-4-hydroxyphenylglycol (MHPG) are the major degradation products of the monoamines dopamine, serotonin and noradrenaline, respectively. Cerebrospinal fluid (CSF) HVA, 5-HIAA and MHPG concentrations are considered to reflect the turnover rates of the monoamines in the central nervous system (CNS). Concentrations of HVA and 5-HIAA in ventricular, cisternal, and lumbar CSF show a craniocaudal gradient [3,4]. Postmortem human studies have shown that CSF HVA and 5-HIAA reflect brain HVA and 5-HIAA concentrations [5,6]. Correlations have been found between MHPG concentrations in CSF and MHPG concentrations in hypothalamus, temporal cortex, and pons in human autopsy cases [6]. Studies in human twins and other primates have shown that monoamine metabolite concentrations are partially under genetic influence [7,8].

Schizophrenia has been associated with monoamine metabolite concentrations, mainly HVA. HVA concentrations have been reported to be significantly lower in drug-free schizophrenic patients compared to controls [9,10]. Both quetiapine and olanzapine administration have been associated with a significant increase in CSF HVA [11,12].

**Figure 1:**
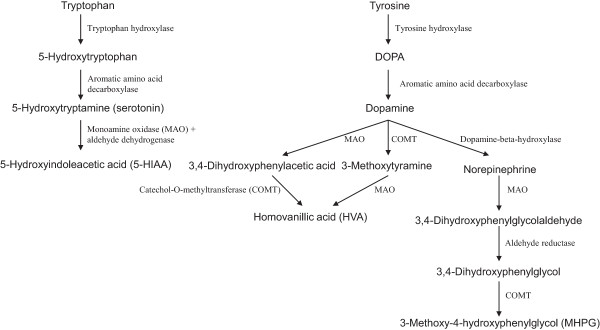
Basic biochemical pathways of serotonin, dopamine and noradrenaline.

Tryptophan hydroxylase (TPH) catalyses the conversion of tryptophan to 5-hydroxytryptophan (Figure 1), which is the rate-limiting reaction in the biosynthesis of serotonin [13]. Two isoforms, TPH1 and TPH2, have been identified, encoded by two different genes, i.e. *TPH1* and *TPH2*, located on chromosome 11p15.3–p14 and 12q21.1, respectively. Both TPH1 and TPH2 are expressed in the human brain and TPH1 may be preferentially expressed in the developing brain [14].

Tyrosine hydroxylase (TH) catalyzes the hydroxylation of L-tyrosine to 3,4-dihydroxy-L-phenylananine (Figure 1), which is the rate-limiting step in the biosynthesis of catecholamines [15]. The *TH* gene is located on chromosome 11p15.5.

DOPA decarboxylase (DDC), also known as aromatic L-amino acid decarboxylase, catalyzes the decarboxylation of both L-DOPA to dopamine and 5-hydroxytryptophan to serotonin [16]. Thus, this enzyme is involved in the dopamine and serotonin pathways and indirectly in the noradrenaline pathway (Figure 1). The *DDC* gene is located on chromosome 7p12.1 and consists of 15 exons spanning more than 75 kb [16].

Dopamine beta-hydroxylase (DBH), localised in vesicles of noradrenergic and adrenergic cells [17], catalyses the conversion of dopamine to noradrenaline (Figure 1). *DBH* knockout mice lack noradrenaline and moreover show attenuated dopamine concentrations in various brain areas, due to interactions between noradrenergic and dopamine systems [18]. The *DBH* gene is located on chromosome 9q34.

Catechol-O-methyltransferase (COMT) catalyses the methyl conjugation and thus inactivation of the catecholamines and is the most important regulator of the prefrontal dopamine function [19]. This enzyme is not involved in the degradation of serotonin (Figure 1). The *COMT* gene is located on chromosome 22q11.2.

Monoamine oxidases are important enzymes for the degradation of various biogenic amines, including catecholamines and serotonin (Figure 1). There are two forms of the enzyme, monoamine oxidase A (MAOA) and monoamine oxidase B (MAOB), located throughout the brain in the outer membrane of mitochondria and encoded by two separate genes [20]. In humans, MAOA preferentially oxidizes serotonin and noradrenaline, whereas MAOB oxidizes dopamine [20]. The *MAOA* and *MAOB* genes are closely linked on the chromosome Xp11.23 and both include 15 exons with identical intron-exon organization, implying that they are derived from the same ancestral gene [21].

In the present study, we considered dopamine, serotonin, and noradrenaline turnover rates in CNS as probable intermediate steps between gene variants and schizophrenia. We hypothesized that single nucleotide polymorphisms (SNPs) in genes encoding the enzymes TPH1, TPH2, TH, DDC, DBH, COMT, MAOA and MAOB, all implicated in the basic biochemical pathways of the monoamines, affect the concentrations of HVA, 5-HIAA, and MHPG in the CSF of psychotic patients.

## Methods

### Subjects

Psychotic patients were initially recruited among inpatients at four psychiatric university clinics in Stockholm County between the years 1973 and 1987. After admittance patients were asked to participate in pharmacological and/or biological research projects as previously described [22]. Patients were usually observed for at least 48 hours without any antipsychotic medication before CSF was drawn by a lumbar puncture.

Three to 34 years after the first investigation, when CSF was obtained, subjects were asked to participate in genetic research and whole blood was drawn for genotyping. Subjects were also asked to participate in a structured interview [23] and to sign a request permitting the researchers to take part of his/her medical records. Available records were scrutinized by a researcher to obtain a life-time diagnosis according to DSM-III-R and DSM-IV. In 2010, 22 to 36 years after the initial CSF sampling, hospital discharge diagnoses were obtained from the Swedish psychiatric inpatient register (SPIR), which is a register based on the patient’s identification number, covering all inpatient hospitalizations in Sweden since 1973. For each hospitalization the diagnosis was recorded according to the International Classification of Diseases, 8^th^, 9^th^ or 10^th^ revisions. The majority of the participating individuals had experienced several hospitalizations, but each patient was given only one diagnosis, following a diagnostic hierarchy as previously described [24,25]. It was not possible to retrieve all medical records and several of the patients were not willing to participate in a diagnostic interview. Therefore, the final diagnoses were based on those obtained from SPIR.

Further analyses, investigating healthy individuals, were conducted for SNPs that were found to be associated with HVA, 5-HIAA or MHPG concentrations in psychotic patients, in order to evaluate whether the effects of the associated SNPs on the specific monoamine metabolites were restricted to patients with psychosis. Unrelated healthy Caucasians were investigated with lumbar puncture between the years 1973 and 1987 to participate in biological research. Eight to 20 years after the first investigation, when CSF was sampled, the subjects were interviewed to re-assess the absence of psychiatric morbidity as previously described [26]. At this interview whole blood was drawn for genotyping.

The study was conducted in accordance with the Declaration of Helsinki and approved by the Ethics Committee of the Karolinska University Hospital. Written informed consent was obtained from all the participating subjects.

### CSF monoamine metabolite concentrations

CSF samples (12.5 ml) were drawn by lumbar puncture with the patients and controls in a sitting or recumbent position between 8 and 9 a.m, after at least 8 h of bed-rest and absence of food intake or smoking. 5-HIAA, HVA, and MHPG concentrations were measured by mass fragmentography with deuterium-labeled internal standards [27]. Back-length was defined as the distance between the external occipital protuberance and the point of needle insertion.

### DNA analysis

Genomic DNA was extracted from whole blood [28]. SNPs in *TPH1* (n = 10), *TPH2* (n = 21), *TH* (n = 10), *DDC* (n = 24), *DBH* (n = 25), *COMT* (n = 16), *MAOA* (n = 6) and *MAOB* (n = 7) were included. These SNPs were either candidate SNPs (n = 28) reported to be associated with schizophrenia, other mental disorders, enzyme function or monoamine metabolite concentrations, or tag-SNPs (n = 91), selected using HapMap to cover the genes of interest with an r^2^ threshold of 0.8. The genotyping was performed using the Illumina BeadStation 500GX and the 768-plex Illumina Golden Gate assay (Illumina Inc., San Diego, CA, USA) [29].

### Statistical analysis

Hardy-Weinberg equilibrium (HWE) was tested using exact significance as implemented in STATA 12.1. Minor allele frequencies were measured using STATA 12.1. Normality of residuals was checked graphically with STATA 12.1. The associations between SNPs and CSF monoamine metabolite concentrations were tested with multiple linear regression (STATA 12.1), where concentration was modeled as a linear function of the allele count (of each SNP separately) and three to five covariates. In the case of psychotic patients, back-length, gender, age at lumbar puncture, diagnosis (i.e. schizophrenia spectrum psychosis or other psychosis) and use of antipsychotics were included as covariates. Antipsychotic treatment was considered as present if the patient had taken antipsychotics during a three-week period prior to the lumbar puncture. In the case of healthy controls, back-length, gender and age at lumbar puncture were included as covariates. *MAOA* and *MAOB* are located on chromosome X, and therefore the analyses of these genes were conducted separately for men and women.

In psychotic patients, we conducted 396 tests in total, as we have included 119 SNPs tested separately for the three different monoamine metabolites and moreover the X-linked *MAOA* and *MAOB* SNPs were tested separately in men and women. Adjustments for multiple testing were performed using Bonferroni correction taking into account a/the total number of tests conducted (α = 0.05/396 = 1.26x10^−4^) and b/ the total number of tests conducted, restricted to the 28 candidate SNPs (α = 0.05/90 = 5.56x10^−4^).

## Results

Totally 74 patients (45 men and 29 women) were included in the present study. Their mean age ± standard deviation was 30.4 ± 7.2 years at lumbar puncture, whereas the mean age of disease onset was 27.6 ± 7.8.

A minority (n = 26) of the patients were treated with antipsychotics at the time of lumbar puncture, whereas the majority (n = 36) were free from antipsychotic medication since three weeks or longer. Sixty-four patients were diagnosed with schizophrenia spectrum disorder (schizophrenia n = 60, schizoaffective disorder n = 4) and ten with other psychosis (psychosis not otherwise specified (NOS) n = 7, delusional disorder n = 1, bipolar disorder n = 1, alcohol induced psychotic disorder n = 1).

We conducted separate analyses, in order to evaluate how the SPIR-derived diagnoses conformed to other diagnostic tools used in the present study. Evaluations were made based on information originating from the medical records in 52 of the patients resulted in a diagnosis of a psychotic disorder in 98% of these individuals. Of 44 patients participating in a diagnostic interview [23], 91% displayed a psychotic disorder according to the SCID-I algorithm. Previous studies have also shown that the Swedish in-patient resister-based diagnoses of schizophrenia spectrum psychosis have a high validity, as 85% to 94% of these patients displayed these diagnoses when research psychiatrists made a diagnostic evaluation using information from medical records and a structured clinical interview [24].

In the 74 psychotic patients, 119 SNPs in eight genes encoding enzymes implicated in the monoamine metabolism were selected and genotyped. The minor allele frequency for the selected markers ranged from 2% to 49%. Departure from HWE (p-value < 0.05) was found in four of the 119 SNPs analyzed, i.e. *DDC* rs4947510, *DDC* rs921451, *TPH2* rs1352250 and *COMT* rs165774. The residuals of the nominal associations were approximately normally distributed. In psychotic patients, the mean (S.D.) concentrations of the three monoamine metabolites were: HVA 178.6 (79.3) nmol/L; 5-HIAA 93.1 (34) nmol/L; MHPG 43.3 (9.3) nmol/L. Twelve, 12 and 18 of the investigated polymorphisms (Tables 1, 2 and 3) were found to be nominally associated with CSF HVA, 5-HIAA and MHPG concentrations, respectively.

**Table 1:** SNPs nominally associated with CSF HVA concentrations in psychotic patients

		**Patients with psychosis (n = 74; 45 men, 29 women)**			**Healthy controls (n = 111; 63 men, 48 women)**		
**HVA mean (S.D.)**		**178.6 (79.3) nmol/l**			**167.5 (68.4) nmol/l**		
**Gene**	**SNP**	**MAF(%)**	**HWE**	***P***	**MAF(%)**	**HWE**	***P***
*DDC*	rs11238133	36	0.211	0.004	41	1.000	0.757
*DDC*	rs6951648	19	0.273	0.005	17	0.735	0.297
*DDC*	rs10499696	11	0.186	0.009	9	0.594	0.681
*DDC*	rs921451	42	0.018	0.011	43	0.564	0.661
*DDC*	rs9942686	21	0.723	0.017	23	1.000	0.305
*TH*	rs10770141	33	0.608	0.023	40	1.000	0.433
*TPH1*	rs211105	31	0.785	0.029	25	0.449	0.292
*TH*	rs10840491	16	0.680	0.035	12	0.640	0.433
*DDC*	rs6593011	15	0.652	0.038	13	0.208	0.959
*COMT*	rs165774	32	0.003	0.043	30	0.258	0.650
*TPH2*	rs1872824	34	0.606	0.047	33	0.830	0.685
*TH*	rs10840489	18	1.000	0.048	14	1.000	0.357

Minor allele frequencies (MAF), p-values of testing for Hardy–Weinberg equilibrium (HWE) and p-values (*P*) from multiple linear regressions of single nucleotide polymorphisms (SNPs) nominally associated with homovanillic acid (HVA) concentrations in the cerebrospinal fluid of psychotic patients and the corresponding association statistics among healthy controls.

**Table 2:** SNPs nominally associated with CSF 5-HIAA concentrations in psychotic patients

		**Patients with psychosis (n = 74; 45 men, 29 women)**			**Healthy controls (n = 111; 63 men, 48 women)**		
**5-HIAA mean (S.D.)**		**93.1 (34) nmol/l**			**90.8 (36.2) nmol/l**		
**Gene**	**SNP**	**MAF (%)**	**HWE**	***P***	**MAF (%)**	**HWE**	***P***
*MAOB*(women)	rs2311013	3	1.000	0.009	4	1.000	0.866
*MAOB*(women)	rs1181252	3	1.000	0.009	4	1.000	0.053
*DDC*	rs9942686	21	0.723	0.009	23	1.000	0.069
*DDC*	rs11238133	36	0.211	0.012	41	1.000	0.516
*DBH*	rs1611118	7	1.000	0.014	7	0.397	0.895
*DDC*	rs11238131	30	0.784	0.019	32	0.826	0.829
*MAOA* (men)	rs5906957	31		0.027	17		0.089
*DDC*	rs11575535	4	1.000	0.035	4	1.000	0.302
*TPH1*	rs1799913	42	0.096	0.039	41	0.334	0.330
*DBH*	rs6271	3	1.000	0.047	5	1.000	0.528
*MAOA* (men)	rs4301558	36		0.048	20		0.123
*MAOA* (men)	rs3027396	36		0.048	21		0.299

Minor allele frequencies (MAF), p-values of testing for Hardy–Weinberg equilibrium (HWE) and p-values (*P*) from multiple linear regressions of single nucleotide polymorphisms (SNPs) nominally associated with 5-hydroxyindoleacetic acid (5-HIAA) concentrations in the cerebrospinal fluid of psychotic patients and the corresponding association statistics among healthy controls. *MAOA* and *MAOB* are located on chromosome X and the analyses were therefore conducted separately for men and women.

**Table 3:** SNPs nominally associated with CSF MHPG concentrations in psychotic patients

		**Patients with psychosis (n = 74; 45 men, 29 women)**			**Healthy controls (n = 111; 63 men, 48 women)**		
**MHPG mean (S.D.)**		**43.3 (9.3) nmol/l**			**41.7 (8.1) nmol/l**		
**Gene**	**SNP**	**MAF(%)**	**HWE**	***P***	**MAF (%)**	**HWE**	***P***
*MAOB* (men)	rs5905512	47		0.0004	50		0.479
*MAOB* (men)	rs1799836	38		0.0008	44		0.483
*DDC*	rs11575535	4	1.000	0.005	4	1.000	0.126
*TPH1*	rs4537731	44	0.475	0.007	40	0.047	0.646
*DDC*	rs6592961	28	0.400	0.008	29	0.104	0.855
*TPH1*	rs7933505	41	0.148	0.010	41	0.334	0.342
*DDC*	rs7809758	40	0.811	0.013	38	0.159	0.956
*TPH1*	rs7122118	43	0.239	0.014	40	0.029	0.821
*DBH*	rs6271	3	1.000	0.017	5	1.000	0.340
*TPH1*	rs1799913	42	0.096	0.017	41	0.334	0.342
*TPH1*	rs684302	42	0.232	0.017	41	0.171	0.397
*DDC*	rs9942686	21	0.723	0.018	23	1.000	0.189
*MAOB* (men)	rs6651806	24		0.021	32		0.328
*DDC*	rs17133853	11	1.000	0.033	9	0.569	0.436
*DBH*	rs1611115	21	1.000	0.035	14	1.000	0.791
*DBH*	rs3025388	18	0.445	0.047	18	0.517	0.064
*TPH1*	rs12292915	44	0.161	0.047	43	0.052	0.066
*TPH1*	rs211105	31	0.785	0.049	25	0.449	0.033

Minor allele frequencies (MAF), p-values of testing for Hardy–Weinberg equilibrium (HWE) and p-values (*P*) from multiple linear regressions of single nucleotide polymorphisms (SNPs) nominally associated with 3-methoxy-4-hydroxyphenylglycol (MHPG) concentrations in the cerebrospinal fluid of psychotic patients and the corresponding association statistics among healthy controls. *MAOA* and *MAOB* are located on chromosome X and the analyses were therefore conducted separately for men and women.

It may be argued that the genes selected for the present report are highly likely to influence monoamine metabolite concentrations in CSF, and giving strong a priori hypotheses, it has been suggested that correction for multiple testing is not necessary [30]. Therefore, we report all the nominal associations. Taking into account the total number of tests conducted, we also applied a Bonferroni correction for multiple testing (α = 0.05/396 = 1.26 × 10^−4^) and none of the nominal associations were found to be statistically significant. As a less conservative alternative, we applied a Bonferroni correction taking into account the total number of tests conducted, restricted to the 28 candidate SNPs (α = 0.05/90 = 5.56x10^−4^). Using this correction, there was one significant result, i.e. the association between MAOB rs5905512 and CSF MHPG concentrations in psychotic men. In total, there were 42 nominal associations, which exceeded the expected number (20) of nominal associations given the 396 calculations performed [(119 SNPs + additional 13 calculations because of the gender-based calculations on the X-chromosome) × 3 monoamine metabolites)].

The associations (n = 42) between SNPs and monoamine metabolite concentrations which gave evidence for nominal significance in psychosis were tested in healthy individuals. There were 111 Caucasians (63 men and 48 women) included for that purpose. Their mean ages ± standard deviation were 28.4 ± 7.5 years at lumbar puncture. In the healthy Caucasians, the mean (S.D.) concentrations of the three monoamine metabolites were: HVA 167.5 (68.4) nmol/L; 5-HIAA 90.8 (36.2) nmol/L; MHPG 41.7 (8.1) nmol/L. The minor allele frequency for the selected markers ranged from 4% to 50%. Departure from Hardy-Weinberg equilibrium (p < 0.05) was found in two of the SNPs analyzed (Table 3). The residuals were approximately normally distributed. With the exception of a nominal association between *TPH1* rs211105 and MHPG concentrations, no associations were found (Table 3).

In the present study, mean CSF HVA and 5-HIAA concentrations were not found to be associated with antipsychotic treatment, whereas mean CSF MHPG concentration was significantly lower in patients who were prescribed antipsychotics compared to antipsychotic-free patients. We have included the use of antipsychotics as a covariate in all our analyses and moreover, our independent variables, i.e. the SNPs, are not expected to be associated with the presence or absence of antipsychotic treatment. Thus, the use of antipsychotics should not confound our analyses, even in the case of MHPG.

## Discussion

In the present study, polymorphisms in genes coding for enzymes of importance for the catecholamine and serotonin metabolism were analyzed. There was an excess of nominally significant associations (42 observed versus 20 expected) among patients with psychotic disorder.

The Hardy-Weinberg principle is used in association studies to detect genotyping errors, inbreeding and population stratification [31]. Deviation from the Hardy-Weinberg proportions in psychotic individuals can provide evidence for association between SNPs and psychosis [31]. In the present study four SNPs showed departure from HWE (p < 0.05) in psychotic patients, i.e. *DDC* rs4947510, *DDC* rs921451, *TPH2* rs1352250 and *COMT* rs165774. Departure from HWE in controls can indicate genotyping errors, inbreeding or population stratification [31]. Lack of HWE may also result as an effect of multiple testing. In the present study, two SNPs showed departure from HWE (p < 0.05) in healthy controls, i.e. *TPH1* rs7122118 and *TPH1* rs4537731. The results regarding these SNPs should be interpreted with caution.

### TPH1 and TPH2

*TPH1* gene variations have been associated with schizophrenia in several studies including meta-analyses (http://www.szgene.org). In the present study, one *TPH1* SNP, i.e. rs1799913, was nominally associated with 5-HIAA concentrations. Rs1799913 has been associated with schizophrenia in single studies including a meta-analysis (http://www.szgene.org). We could therefore hypothesize that the previously reported association between *TPH1* rs1799913 and schizophrenia may be mediated by serotonergic mechanisms and altered serotonin turnover rate in CNS.

One and seven *TPH1* SNPs were associated with HVA and MHPG concentrations, respectively. Four of the polymorphisms associated with MHPG concentrations, including rs1799913, have previously been reported to be associated with schizophrenia (http://www.szgene.org).

One *TPH2* SNP has been associated with the severity of positive psychotic symptoms in patients with schizophrenia [32]. In the present study *TPH2* rs1872824 was found to be associated with CSF HVA, whereas no association was found between *TPH2* SNPs and CSF 5-HIAA or MHPG concentrations.

### TH

*TH* gene variants have been associated with schizophrenia in 6 out of 19 studies. However, in a meta-analysis no association could be confirmed (http://www.szgene.org). Moreover, *TH* gene variation has been reported to be associated with CSF HVA and MHPG concentrations in healthy controls [33].

In the present study three *TH* SNPs, i.e. rs10770141, rs10840491 and rs10840489, were associated with HVA concentrations in patients with psychosis. Rs1077014, located upstream of *TH* in the proximal promoter, affects the transcriptional activity of the promoter and the catecholamine secretion in humans [34]. Rs1077014 may be associated with persistence, a typical personality trait in chronic fatigue syndrome patients [35] as well as with the personality trait of novelty seeking in healthy men [36]. To our knowledge rs10840491 and rs10840489 have not been ascribed any functionality or association with schizophrenia or other mental disorders.

### DDC

No studies have shown significant associations between *DDC* SNPs and schizophrenia (http://www.szgene.org), whereas an association between *DDC* genotypes and age at disease onset has been found in men with schizophrenia [37]. Moreover, *DDC* deficiency has been reported to result in decreased HVA and 5-HIAA concentrations in CSF [38,39].

In the present study six, four and five *DDC* SNPs, all intronic, were nominally associated with CSF HVA, 5-HIAA and MHPG concentrations, respectively. Rs6592961 has been reported to be significantly associated with autism [40]. In the present study, this SNP was nominally associated with MHPG concentrations in psychotic patients.

### DBH

In the present study, two *DBH* SNPs, i.e. rs1611118 and rs6271 and three *DBH* SNPs, i.e. rs6271, rs1611115, and rs3025388 were found to be nominally associated with 5-HIAA and MHPG concentrations, respectively, in psychotic patients. Rs1611115, located upstream of *DBH,* accounts for 31% to 52% of the variance of plasma DBH activity in different human populations [41], whereas rs6271, a nonsynonymous SNP located in exon 11, independently accounts for additional variance [42].

*DBH* SNPs have been associated with schizophrenia in 2/17 studies conducted (http://www.szgene.org). Rs1611115 has been significantly associated with attention-deficit hyperactivity disorder in children [43], as well as with impulsiveness and aggressive hostility in adults [44]. Moreover, this SNP has been found to be associated with alcohol dependence in women [45]. Rs6271 was nominally associated with schizophrenia in both case–control and family based analyses in a north Indian schizophrenia cohort [46] and significantly associated with bipolar disorder in a Turkish population [47]. Rs1611118 and rs3025388 are intronic and to our knowledge, have not been ascribed any functionality or association with mental disorders.

### COMT

*COMT* is a candidate gene for schizophrenia with many studies, including meta-analyses, reporting association between gene polymorphisms and the disorder (http://www.szgene.org). *COMT* gene variations have not shown associations with monoamine metabolite concentrations in healthy controls or in a mixed group of psychiatric patients [48,49]. In the present study, the intronic *COMT* rs165774 was nominally associated with HVA concentrations. Rs165774 showed departure from Hardy-Weinberg equilibrium in psychotic patients but not in healthy controls. Rs165774 has been reported to be significantly associated with schizophrenia [50] and alcohol dependence [51].

### *MAOA* and *MAOB*

A minority of studies searching for association between *MAOA* or *MAOB* gene variation and schizophrenia reported a relationship (2/29 and 3/15, respectively) (http://www.szgene.org). *MAOA* polymorphisms have been associated with CSF HVA concentrations in patients with atypical depression [52], alcohol dependence and controls [53,54] as well as in a mixed group of psychiatric patients [49]. *MAOA* polymorphisms have also been associated with 5-HIAA in healthy men [55] and women [54].

In the present study, three *MAOA* SNPs were associated with 5-HIAA concentrations in psychotic men (Table 2), whereas three *MAOB* SNPs were associated with MHPG concentrations in psychotic men (Table 3) and two *MAOB* SNPs were associated with 5-HIAA concentrations in psychotic women (Table 2).

The associations between *MAOB* rs5905512 and *MAOB* rs1799836 and MHPG concentrations displayed the lowest p-values in the present study. *MAOB* rs5905512 was associated with MHPG concentrations in psychotic men (uncorrected p = 0.0004), which is statistically significant taking into account only the candidate SNPs tested. No association was found between rs5905512 and psychotic women, healthy men or healthy women. To our knowledge, two studies have searched for association between this SNP and schizophrenia (http://www.szgene.org) and our results are in accordance with one of these two studies [56], reporting significant association between rs5905512 and schizophrenia only in men, whereas the other study did not find any associations in either gender [57]. Moreover, rs5905512, located in intron 1, is a perfect proxy of a haplotype spanning from intron 1 to intron 3 and being subject to recent selection, in agreement with the ancestral susceptibility hypothesis of schizophrenia [56]. *MAOB* rs1799836 was also associated with MHPG concentrations in men with psychosis (uncorrected p = 0.001) in the present study. Rs1799836 is located in intron 13 and is associated with altered enzyme activity in vitro and in vivo [58,59]. Rs1799836 has been associated with schizophrenia in women [60]. The third *MAOB* SNP that was found to be associated with MHPG concentrations in psychotic men, i.e. rs6651806, has previously been reported to be associated with negative emotionality in healthy individuals [61]. To our knowledge, no functionality or association with mental disorders has been ascribed to the other *MAOA* (rs5906957, rs4301558, rs3027396) and *MAOB* (rs2311013, rs1181252) variants showing nominal association with monoamine metabolites in the present study.

### Limitations

The present study has certain limitations. The relatively small number of participants, both patients and controls, in association with the large number of tests conducted, results in a limited power to detect possible associations between SNPs and CSF monoamine metabolite concentrations after correction for multiple testing. Moreover, the non-inclusion of tobacco use as a covariate may influence the results, especially with regard to HVA concentrations [62]. Finally, the associations found in the present study need replications in independent studies.

## Conclusions

In psychotic patients, we found nominal associations between SNPs in genes encoding enzymes implicated in the monoamine pathways and the CNS monoamine turnover rates, as reflected by the CSF concentrations of HVA, 5-HIAA and MHPG. There were 42 nominal associations, which exceed the expected number (20) of nominal associations given the total number of tests performed. Forty-one out of these 42 suggestive associations were restricted to patients with psychosis and were absent in healthy controls. Some of the associated SNPs have been reported to be associated with schizophrenia. Moreover, one candidate *MAOB* SNP, previously reported to be associated with schizophrenia in men, was found to be significantly associated with MHPG concentrations in men with psychotic disorder, performing a correction for multiple testing for the number of candidate SNPs selected. Taken together, the present study supports the notion that altered monoamine turnover rates reflect intermediate steps in the associations between gene variations and psychosis.

## Abbreviations

HVA: Homovanillic acid; 5-HIAA: 5-hydroxyindoleacetic acid; MHPG: 3-methoxy-4-hydroxyphenylglycol; CSF: Cerebrospinal fluid; CNS: Central nervous system; TPH: Tryptophan hydroxylase; TH: Tyrosine hydroxylase; DDC: DOPA decarboxylase; DBH: Dopamine beta-hydroxylase; COMT: Catechol-O-methyltransferase; MAOA: Monoamine oxidase A; MAOB: Monoamine oxidase B; SNP: Single nucleotide polymorphism; SPIR: Swedish psychiatric inpatient register; HWE: Hardy-Weinberg equilibrium.

## Competing interests

The authors declare that they have no competing interests.

## Authors’ contributions

DA contributed to the conception and design of the study, participated in subject assessment, subject characterization and the statistical analysis, managed the literature search and web-based database searches and drafted the article. ES performed the statistical analysis. TA was in charge of the genotyping procedures. GCS made a contribution to the conception and design of the study and to the acquisition of data. LT and IA contributed to the conception and design of the study. EGJ contributed to the conception and design of the study, the acquisition and the interpretation of data. All authors revised the article critically for important intellectual content and approved the final manuscript.
